# *Si-ni-tang* (a Chinese herbal formula) for improving immunofunction in sepsis: study protocol for a pilot randomized controlled trial

**DOI:** 10.1186/s13063-019-3646-3

**Published:** 2019-08-28

**Authors:** Ruifeng Zeng, Yi Zheng, Rongrong Fan, Gengbiao Zhou, Yan Zhang, Shutao Mai, Dongping Xie, Yanna Weng, Jiongdong Du, Yun Han, Fang Lai

**Affiliations:** 1 The Second Affiliated Hospital of Guangzhou University of Chinese Medicine, Guangzhou, Guangdong China; 2 Guangdong Provincial Hospital of Chinese Medicine, Guangzhou, Guangdong China; 3 Doctoral student of Guangzhou University of Chinese Medicine, Guangzhou, Guangdong China; 4 Foshan Hospital of Traditional Chinese Medicine, Foshan, Guangdong China; 5 Guangdong Provincial Key Laboratory of Research on Emergency in TCM, Guangzhou, Guangdong China

**Keywords:** Sepsis, immune function, Protocol, *Si-ni-tang* (SNT), Chinese herbal medicine

## Abstract

**Background:**

Immunologic derangement may be the critical pathophysiologic mechanism in sepsis, and immunotherapy might be a potential new treatment. *Si-ni-tang* (SNT), an ancient Chinese herbal formula documented in *Shanghan Lun*, has been used for treating severe sepsis for thousands of years. Research shows that it may have a therapeutic benefit for sepsis. This study will evaluate the feasibility of testing the effects of SNT on immune function in sepsis patients.

**Methods/design:**

This is a pilot randomized controlled study. Eligible sepsis patients admitted to our medical intensive care unit will be randomly allocated to the control group or the SNT group. Both groups will receive standard therapy according to the recommendations of the Surviving Sepsis Campaign. In addition, the SNT group will receive SNT (150 mL per day for 3 days) orally or by gastric tube, while the control group will receive 150 mL of normal saline. The primary outcome is to assess the feasibility of this treatment. The secondary outcomes include: (1) immune function measured by monocyte human leukocyte antigen-DR (mHLA-DR) expression, procalcitonin, and the ratio of CD4+ to CD8+ T lymphocytes and (2) other clinical data, such as the 28-day all-cause mortality, Sequential Organ Failure Assessment (SOFA) scores, Acute Physiology and Chronic Health Evaluation (APACHE) II scores, both of the latter on days 0 and 3.

**Discussion:**

This study aims to evaluate the feasibility of testing the efficacy of SNT for treating sepsis when used as an adjunctive treatment with the standard therapy recommended by the Surviving Sepsis Campaign.

**Trial registration:**

ClinicalTrials.gov, NCT02777606. Registered on 22 June 2016. Retrospectively registered. https://clinicaltrials.gov/

**Electronic supplementary material:**

The online version of this article (10.1186/s13063-019-3646-3) contains supplementary material, which is available to authorized users.

## Background

Sepsis is a systemic clinical syndrome caused by infectious disease. It is still a major global health problem due to its high incidence and mortality rates, even though supportive care has improved significantly.

During an infection, the immune system is activated to eliminate the pathogen. In sepsis, very often the immune system is over-activated and excessive tissue damage occurs, which results in multi-organ dysfunction [1]. However, studies focused on blocking the hyper-inflammatory response have shown no therapeutic benefit. Indeed, in some cases, this treatment has impacted outcomes negatively [2, 3]. One of the reasons for this failure may be that immune suppression occurs soon after sepsis initiates [4], although immunosuppression is much more frequently observed in the late phase of sepsis. Studies have indicated that both innate and adaptive immunity are suppressed in sepsis, leading to a profoundly decreased production of either pro- or anti-inflammatory cytokines [5, 6], a severe loss of immune cells [7], the downregulation of HLA-DR [6], and impairment of the function of all types of surviving immune cell [5]. Moreover, blocking the hyper-inflammatory response results in an inability to eliminate the pathogen. Therefore, achieving a balance between the hyper- and hypo-inflammatory phases of the immune response against invading pathogens may be a promising treatment for sepsis [8].

*Si-ni-tang* (SNT) consists of *Aconitum carmichaelii Debeaux*, *Zingiber officinale Roscoe*, and *Glycyrrhiza uralensis Fisch*. It was first described in the *Shanghan Lun*, the oldest monograph on infectious diseases, dating back almost 2000 years. SNT is a classical herbal formula prescribed to recuperating patients after a collapse [9], which is a common syndrome observed in sepsis [10]. Furthermore, SNT has been reported to alleviate inflammatory response [11, 12], ameliorate microcirculatory disturbances [13], and improve shock reversal [14] in sepsis in people and animals. However, the effects of SNT on immune function in sepsis have not been comprehensively investigated.

Therefore, the present pilot, a randomized controlled study, has been designed to evaluate the feasibility of using SNT to improve the immune function of sepsis patients.

## Methods/design

### Aim

The study is a prospective randomized controlled pilot trial designed to evaluate, for sepsis patients, the feasibility of testing the effects on immune function of SNT administration in addition to the standard therapy recommended by the Surviving Sepsis Campaign.

### Design

**Fig. 1:**
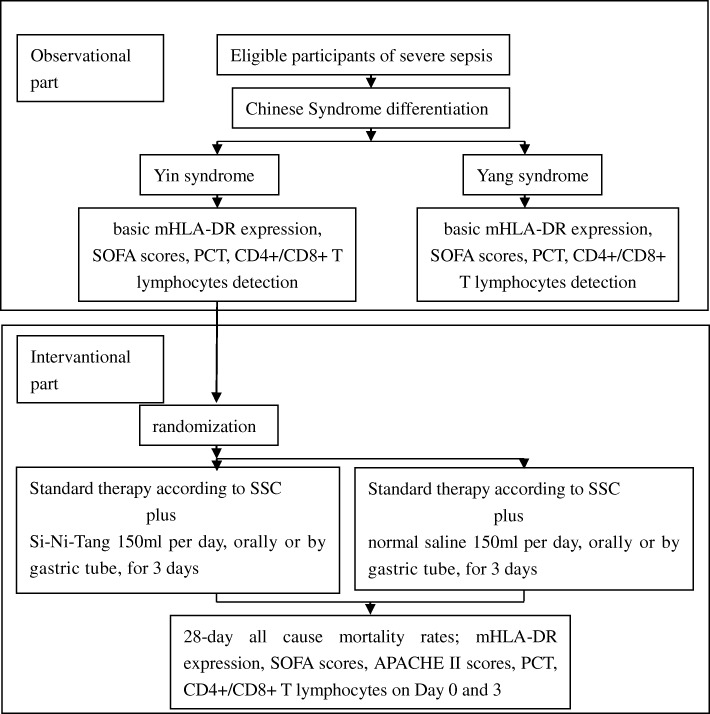
Study flow chart. mHLA-DR monocyte human leukocyte antigen-DR, SOFA Sequential Organ Failure Assessment, APACHE Acute Physiology and Chronic Health Evaluation, PCT procalcitonin, SSC Surviving Sepsis Campaign

This interventional study will be carried out in the intensive care unit (ICU) of Guangdong Provincial Hospital of Chinese Medicine. It is embedded within an observational study of the immune function of patients with severe sepsis. The trial has received ethical approval from the institutional review board of Guangdong Provincial Hospital of Chinese Medicine (approval number B2016–010.2-01). The investigation will conform to national laws and the Declaration of Helsinki. Eligible participants or their authorized representatives will provide written informed consent before randomization. The protocol adheres to the Recommendations for Interventional Trials (SPIRIT) guidelines (Additional file 1). Figure 1 is a flow chart of the study. If any significant changes must be made to the protocol, a draft of the new version will be submitted for approval from the institutional review board of Guangdong Provincial Hospital of Chinese Medicine.

Participants can withdraw from the trial for any reason at any time. Researchers can remove participants from the trial to ensure their safety or to maintain the quality of the trial. Participants meeting any of the following conditions may need to be removed from the study: (1) those with severe complications or a deterioration of general health, (2) those violating the study protocol, or (3) those who are lost to the follow-up.

### Eligibility criteria

Patients who meet the inclusion criteria of sepsis are currently being enrolled in the observational study, which is evaluating basic immune functions. In addition, those who have a diagnosis of *yin* syndrome according to the principles of syndrome differentiation in traditional Chinese medicine made by two associate chief physicians or above are being enrolled into the embedded interventional study. Any disagreement will be resolved after discussion with a third party YH.

#### Inclusion criteria

The inclusion criteria for the observational study [15] are as follows:

1. Aged between 18 and 85
2. Documented or suspected infection
3. Two of the following induced by the infection:
  - rectal temperature between 38 °C and 36 °C
  - heart rate >90 beats per minute
  - respiration rate >20 breaths per minute, partial pressure of carbon dioxide in arteries pa CO_2_ < 32 mmHg, or requiring ventilation
  - white blood cell count >12 × 10^9^ L^-1^ or <4 × 10^9^ L^-1^, or immature forms of white blood cells >10%
4. One of the following:
  - arterial hypotension (systolic blood pressure, SBP < 90 mmHg, mean arterial pressure <65 mmHg, or SBP decrease >40 mmHg in adults or vasopressor-dependent to maintain SBP ≥ 90 mmHg or mean arterial pressure ≥ 65 mmHg) persisting for 1 hour or more in spite of adequate fluid resuscitation
  - oxygenation index ≤300
  - pH ≤ 7.30 or base excess ≤ –5.0 mmol L^-1^ with hyperlactatemia (≥3 mmol L^-1^)
  - acute oliguria (urine output <0.5 mg kg^-1^ h^-1^ for at least 2 hours), creatinine increase >2 mg dL^-1^ within 48 h, or requiring renal replacement therapy
  - hyperbilirubinemia (plasma total bilirubin > 2 mg/dL or 35 μmol L^-1^)
  - platelet count < 10 × 10^12^ L^-1^
  - international normalized ratio >1.5 or activated partial thromboplastin time >60 s

The inclusion criteria for the interventional study are as follows:

1. Meet all the criteria for the observational part
2. *Yin* syndrome, presenting some of the following [16]:
  - sudden drop of body temperature or cold extremities
  - fatigue
  - pale complexion
  - dull pain
  - cold sweat or sticky sweat
  - dull purplish or pale tongue
  - faint or deep slow pulse

#### Exclusion criteria

The exclusion criteria for the interventional study are as follows:

1. Pregnant or lactating women
2. Patients who received immunosuppressive or immunoenhancement therapy within the past 3 months
3. Patients with a known or suspected autoimmune disease
4. Patients not expected to survive 28 days due to end-stage disease or other uncorrectable medical condition
5. Fasting subjects
6. Known or suspected allergy to any ingredient of SNT

### Randomization

The randomization sequence was created in SPSS 17.0 with a block randomization size of four by a physician (RZ) not involved in delivering the treatment or the follow-up assessment. The block randomization is to ensure the two groups have the same number of participants, as far as is possible. The assignment is balanced, with two participants for each group in each block. The randomization block size will be concealed. The allocation codes are then placed in sealed opaque envelopes until participants are randomized.

At least one researcher in the study group will be notified whenever a potential participant is admitted to the ICU. Y Zhang and SM will enroll participants and randomly assign them to either the SNT group or the control group in a 1:1 ratio according to the prepared allocation sequence. A unique code will be assigned to each participant, which will be stored securely by the study group. For each participant, there is an equal probability of being assigned to either group, and allocation cannot be influenced by the researchers.

### Blinding and masking

The laboratory technicians and the biostatisticians responsible for the statistical analysis will be blinded to the assigned treatments. They will not be unblinded under any circumstance.

### Sample size determination

There are no previous clinical studies on using SNT to improve immune function in sepsis, and because of the pilot nature of this study, we need to recruit at least 20 participants in each group to achieve sufficient precision for a sample size calculation in a subsequent full trial [17]. With a 20% dropout rate, we plan to recruit a total of 50 participants. Moreover, since participants can withdraw or may be removed from the trial, 52–60 random numbers may ultimately be needed. Thus, the final number of participants in each group may be differ.

### Interventions and procedures

The study is conducted in the ICU of Guangdong Provincial Hospital of Chinese Medicine. The SNT decoction comprises 15 g of *Aconitum carmichaelii Debeaux* (*shufuzi*), 9 g of *Zingiber officinale Roscoe* (*ganjiang*), and 6 g of *Glycyrrhiza uralensis Fisch* (*zhigancao*). It is manufactured by the Pharmaceutical Department of Chinese Herbal Medicine of Guangdong Provincial Hospital of Chinese Medicine, which stores the medication until it is needed. To make SNT, the three ingredients are immersed separately in 500 mL of distilled water. Next, the *shufuzi* is boiled for 30 min. The remaining two ingredients are then added, boiled, and condensed down to 150 mL.

Consenting eligible patients will be randomized to receive 150 mL of SNT orally per day for 3 days (SNT group) or the equivalent volume of normal saline (control group, to equalize the effect of fluid supplement in severe sepsis) orally or by gastric tube. In addition, all participants will receive standard therapy according to the international guidelines of the Surviving Sepsis Campaign [1]. Immunosuppressive and immunoenhancement therapy are prohibited.

The investigators will be trained beforehand and provided with a printed standardized protocol and case report form. Implementation of the trial will be monitored at least once every 2 weeks by YW and JD, who not involved in enrollment of participants or the data analysis.

### Mechanism of action

*Shufuzi* can form *yang*. It is the “sovereign” ingredient. It is pungent and sweet, and it warms the kidneys. *Ganjiang* is pungent and warm. It is the “minister” ingredient. It can restore *yang* when combined with *shufuzi*. *Zhigancao* reduces tension and alleviates the dryness-heat effects of *shufuzi* and *ganjiang.* It is the “assistant” ingredient. This combination of the three herbs can prevent the collapse of *yang*. Due to the long-term experience and its extensive clinical application, the SNT decoction used in our trials is considered safe and effective for patients with septic shock. However, according to the principles of modern clinical trials, its use has been criticized due to a lack of adequate assessment. Thus, to evaluate the feasibility of testing the efficacy of SNT administration, we designed this randomized controlled pilot trial.

### Standard therapy

Standard therapy is based on current recommendations [1], including antimicrobial therapy, initial resuscitation, fluid therapy, mechanical ventilation, glucose control, sedation or analgesia, renal replacement therapy, and adequate nutrition. Empiric broad-spectrum antimicrobial therapy is administered as soon as sepsis is diagnosed. The optimal dose and the route of administration follow medical standards. In addition, a daily assessment to allow a de-escalation of antimicrobial therapy is recommended. Protective lung ventilation is administered if acute respiratory distress syndrome occurs due to the sepsis, which is defined as a tidal volume of 6–8 mL/kg (predicted body weight), platform pressure <30 cm H_2_O, positive end-expiratory pressure ≥5 cm H_2_O, minimum tolerated oxygen concentration (FiO2), and oxygen saturation of 88–92%.

### Outcomes

The primary endpoint is the feasibility of the study. It will (1) explore the feasibility of recruiting, randomizing, and retaining participants, (2) evaluate the acceptability and compliance of SNT as an intervention alongside usual care, (3) evaluate the appropriateness of secondary outcome measures, (4) generate data for effect size calculations for future trials, and (5) be used to develop an appropriate protocol for further trials. The time required to recruit 50 participants, the recruitment rate, and the dropout rate will be measured. Successful recruitment is defined as at least half (50%) of eligible patients enrolled, with a dropout rate of no more than 20%.

The secondary endpoints include:

- 28-day all-cause mortality rates
- immune function data: monocyte human leukocyte antigen-DR (mHLA-DR) expression and ratio of CD4+ to CD8+ T lymphocytes
- inflammation data: CRP, interleukin-6 (IL-6), interleukin-10 (IL-10), procalcitonin, and tumor necrosis factor α (TNF-α) levels
- Sequential Organ Failure Assessment (SOFA) scores
- Acute Physiology and Chronic Health Evaluation (APACHE) II scores

The exploratory outcomes are mortality in the ICU, length of stay in the ICU, hospital mortality, and length of hospital stay. Safety outcomes include a complete blood count, a general urine analysis, C-reactive protein (CRP), liver function tests (alanine transaminase (ALT), aspartate transaminase (AST), alkaline phosphatase (ALP), total bilirubin (TBIL), and gamma-glutamyltransferase (GGT)), and a renal function test (blood urea nitrogen (BUN) and serum creatinine (Scr)), all of which will be performed at days 0 and 3.

### Data acquisition and assessment of biological data

Participants receive a study ID, which is used to conceal their identity in documents. Investigators involved in this study have access to their actual identity only on an as-needed basis.

The recruitment rate is the percentage of eligible patients who agree to participate in the study. The dropout rate is the percentage of patients who are lost after enrollment up to the 28th day from enrollment, after which no outcome data are collected. Follow-ups are by bedside visit if the patient is still in hospital, or by phone call when a face-to face follow up is not available.

**Fig. 2:**
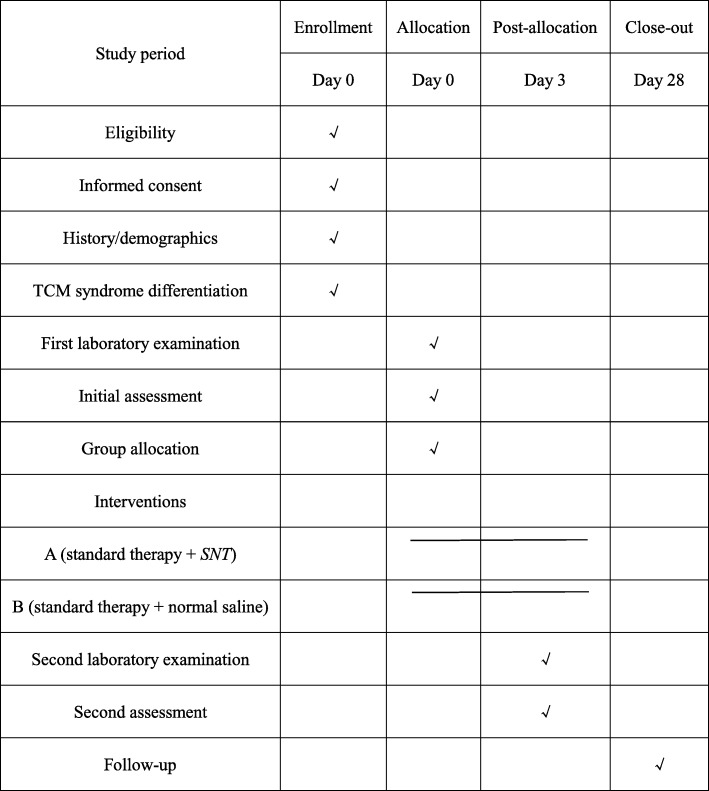
Standard Protocol Items: Recommendations for Interventional Trials (SPIRIT) timeline of measurements. Laboratory examination includes the immune function data (mHLA-DR expression, ratio of CD4+ to CD8+ T lymphocytes), inflammation data (C-reactive protein, interleukin-6, interleukin-10, PCT and TNF-α), complete blood count (CBC), general urine analysis, liver function tests (ALT, AST, ALP, TBIL, and GGT), renal function test (BUN and Scr). ALT alanine transaminase, ALP alkaline phosphatase, AST aspartate transaminase, BUN blood urea nitrogen, CBC complete blood count, GGT gamma-glutamyltransferase, mHLA-DR monocyte human leukocyte antigen-DR, PCT procalcitonin, TBIL total bilirubin, TNF-α Tumor necrosis factor α, Scr serum creatinine,SNT Si-Ni-Tang,SPIRIT Standard Protocol Items: Recommendations for Interventional Trials

As well as the outcome measures, the following clinical data will be collected from all participants: age, gender, infection source, and co-morbidities. Immune function data, inflammation data, APACHE II scores, and SOFA scores will be collected on the day of enrollment and 3 days after enrollment. The trial schedule is listed in Fig. 2.

All mHLA-DR measurements are done in the central laboratory of Guangdong Provincial Academy of Chinese Medical Sciences by flow cytometry (FC-500; Beckman Coulter, Inc., USA) within 4 h after the blood draw. First, 100 μL of unprocessed ice-stored peripheral whole blood sample (anticoagulated with EDTA-K2) will be added to 10 μL of HLA-DR-FITC (clone L243, eBioscience, Inc. SanDiego, CA, USA), CD14-PE (clone 61D3, eBioscience, Inc., San Diego, CA, USA) and CD45-PC5 (clone HI30, eBioscience, Inc. SanDiego, CA, USA) separately. Negative control antibodies are used as follows: Mouse IgG1 K Isotype Control PE (eBioscience, Inc. SanDiego, CA, USA), Mouse IgG1 K Isotype Control PE-Cyanine5 (eBioscience, Inc. SanDiego, CA, USA) and Mouse IgG2a K Isotype Control FiTC (eBioscience, Inc. SanDiego, CA, USA). CD14+ monocytes will be characterized, and 2000–5000 monocytes will be collected. The number of HLA-DR+ monocytes out of the total monocyte population will be recorded as a percentage.

IL-6, IL-10, and TNF-α will be measured with an additional 2–3 mL of peripheral venous blood using ELISA kits (Cusabio, CSB-E04638h; Cusabio, CSB-E04593h; Cusabio, CSB-E04740h). The serum will be separated by centrifugation at 3000 rpm for 20 min and stored at −80 °C for the final analysis. Repeated freezing and thawing will be avoided.

Routine laboratory examinations of complete blood counts, general urine analysis, CRP, liver function tests (ALT, AST, ALP, TBIL, and GGT), and renal function tests (BUN and Scr) will be done by the clinical laboratory of Guangdong Provincial Hospital of Chinese Medicine.

Researchers are trained to collect trial data carefully according to a standard protocol. Molecular biomarkers will be measured in triplicate to ensure quality. All data will be input into an electronic database (REDCap 5.7.0, hosted at Peking Union Medical College Hospital) designed for the study. The data will be entered independently into the database by two researchers and will be checked separately by different trained researchers. All collected data forms will be kept in the hospital’s Scientific Research Department.

All adverse events after enrollment to the 28th day after enrollment, regardless of whether they are related to the intervention, will be recorded. All severe adverse events and unexpected adverse events will be reported to the ethics committee within 1 day. The intervention will be suspended if necessary. Symptomatic treatment will be offered when needed. The principal investigator will decide whether patients affected by adverse events should discontinue their participation.

Data and safety will be monitored by the institutional review board of Guangdong Provincial Hospital of Chinese Medicine and the Scientific Research Department of Guangdong Provincial Hospital of Chinese Medicine, which are independent of the sponsor and have no competing interests.

### Statistical analysis

Quantitative numerical data with a normal distribution will be presented as mean ± standard deviation, while data with a non-normal distribution will be presented as median and interquartile range. Categorical variables will be expressed as frequencies or numbers. Equality of variances between groups will be assessed by Levene’s test. An unpaired Student’s *t*-test will be used for normally distributed quantitative variables, while the Wilcoxon nonparametric statistic will be used for non-normally distributed variables. Pearson’s chi-squared test or Fisher’s exact test will be used for categorical data when appropriate. Differences in biological metrics between arms over time will be evaluated by repeat measures of a general linear model, if appropriate. An intention-to-treat analysis will be used for data for participants who did not complete the study or the follow-ups after treatment.

*P <* 0.05 will be considered as statistically significant. All statistical analyses will be performed with SPSS (version 17.0, Chicago, USA) for Windows by researchers not involved in implementing the trial.

## Discussion

Sepsis is a significant cause of ICU admissions and still the leading cause of death in an ICU [18–22]. It has been generally established that the dysregulated host response induced by infection is the core problem of sepsis [1]. Besides controlling the infection focus and offering support to dysfunctional organs, regulating the comprehensive immune disorders has been an active research topic for several decades. When sepsis initiates, both pro- and anti-inflammatory responses occur. Moreover, a few days later, defects develop in immunity, which become evident in patients with unresolved septic problems [4]. With the many failures in clinical studies to block the hyper-inflammatory response during sepsis, the correlation between immunosuppression and increased risk of infections and death has gained increased interest [23].

Both innate and adaptive immunity are suppressed in sepsis, ranging from the production of both pro- and anti-inflammatory cytokines, to reduced immune cells counts and function [5–7]. Among the reported immune alterations, few have been linked with better clinical outcomes. However, the levels of mHLA-DR may be a reliable indicator for estimating immunosuppression and the severity of sepsis [24, 25]. The change in mHLA-DR over time may be a reliable marker for predicting mortality in sepsis patients [26]. Moreover, mHLA-DR can also be used to assess the effect of immunomodulatory therapies in sepsis [23].

According to Western medicine, there are no specific clinical signs or symptoms of immunosuppression. However, according to Chinese medicine, immunosuppression can be ascribed to *yin* differentiation, while a pro-inflammatory response can be ascribed to *yang* differentiation. Moreover, sepsis patients can also be ascribed to *yin* or *yang* differentiation using clinical information. The observational part of the trial could provide data on whether the *yin zheng* from the molecular level is related to the *yin zheng* from the clinical level.

SNT, a classical herbal formula, has been used for the collapse stage of severe diseases including sepsis in China for thousands of years. It restores *yang* and its warming effect dispels cold [9]. It may have therapeutic effects in patients and animals with sepsis, such as alleviating an inflammatory response [11, 12], ameliorating microcirculatory disturbances [13], and improving shock reversal [14]. However, the effects of SNT on immune function in sepsis have never been investigated. To our knowledge, the present pilot study is the first clinical trial to explore the potential underlying molecular mechanisms and changes in immune function in sepsis due to SNT. The results of this pilot trial may provide insights into the feasibility of a large-scale trial on SNT applied as an adjunctive immunostimulating therapy for a specific subgroup of sepsis patients.

There are several limitations to this pilot study. First, the trial does not have an oral placebo control. When the experimental drug is in the form of granules and powders, an oral placebo can be produced with the same appearance and odor. However, a placebo decoction prepared with distilled water and food coloring to resemble SNT would not have the same smell and taste as the experimental decoctions used in this study. Also, *zhigancao*, a commonly used herb, could not be boiled and used as a placebo decoction alone, since it has a specific pharmacological effect in restoring *qi*. Hence, there is no oral placebo.

Second, this is a pilot trial with a small sample size. The participants are not blinded, and the treatment sepsis patients received is complicated. Hence, this study may not be able to find a significant therapeutic effect.

### Trial status

We are currently recruiting participants.

## Additional file

Additional file 1 SPIRIT 2013 checklist. (DOCX 40 kb)

## Data Availability

Documents on ethics approvals, consent, and funding are available for review by the editorial office. FL and YH are responsible for the data and will be custodians of the final dataset. The results will be reported to Guangdong Science and Technology Department and made available to the public. The investigators plan to publish the results in journals. Participants may request information on study progress and related information.

## Abbreviations

ALP: Alkaline phosphatase
ALT: Alanine transaminase
APACHE: Acute Physiology and Chronic Health Evaluation
AST: Aspartate transaminase
BUN: Blood urea nitrogen
CBC: Complete blood count
CRP: C-reactive protein
GGT: Gamma-glutamyltransferase
HLA-DR: Human leukocyte antigen-DR
ICU: Intensive care unit
IL-10: Interleukin-10
IL-6: Interleukin-6
mHLA-DR: Monocyte human leukocyte antigen-DR
PCT: Procalcitonin
Scr: Serum creatinine
SBP: Systolic blood pressure
SNT: *Si-ni-tang*
SOFA: Sequential Organ Failure Assessment
SPIRIT: Standard Protocol Items: Recommendations for Interventional Trials
TBIL: Total bilirubin
TNF-α: Tumor necrosis factor α
