# Supplementary material for: Si-ni-tang (a Chinese herbal formula) for improving immunofunction in sepsis: study protocol for a pilot randomized controlled trial
Source: Trials. 2019 Aug 28;20:537. doi: 10.1186/s13063-019-3646-3 (PMC6714400; doi:10.1186/s13063-019-3646-3)
Supplement: Supplementary file 1 — SPIRIT 2013 checklist. (DOCX 40 kb) [file 13063_2019_3646_MOESM1_ESM.docx]

**Additional file 1:SPIRIT Checklist: Recommended Items to Address in a Clinical Trial Protocol and Related Documents**

| **Section/Item**  **Administrative information** | **Item Number** | **Description** |
| --- | --- | --- |
| Title | 1 | Descriptive title identifying the study design, population, interventions, and, if applicable, trial acronym  *Si-Ni-Tang* (a Chinese herbal formula) for improving immunofunction in sepsis: study protocol for a pilot randomized controlled trial (P1) |
| Trial registration | 2a | Trial identifier and registry name. If not yet registered, name of intended registry.  Trial identifier：ClinicalTrials.gov: NCT02777606 (P2)  Registry name: The Efficacy of *Si-Ni-Tang* (a Chinese Herbal Formula) for Severe Sepsis |
|  | 2b | All items from the World Health Organization Trial Registration Data Set (Appendix Table, available at [www.annals.org](http://www.annals.org))  Not applicable for this pilot trial. |
| Protocol version | 3 | Date and version identifier  Date July 1, 2016 and version 2.0 |
| Funding | 4 | Sources and types of financial, material, and other support  This work was financially supported by Guangdong Science and Technology Projects, China (2014A020212280 and 2016A020215196); the Natural Science Foundation of Guangdong Province, China (2017ZC0133); the National Natural Science Foundation of China (81703856). (P16) |
| Roles and responsibilities | 5a | Names, affiliations, and roles of protocol contributors  Rui-Feng Zeng^1,2,3^ , Yi Zheng^4^, Rong-Rong Fan^3^, Geng-Biao Zhou^1,2^, Yan Zhang^1,2^, Shu-Tao Mai^1,2^, Dong-Ping Xie^1,2^, Jiong-Dong Du^1,2^, Yan-Na Weng^1,2^, Jing-Xia Zheng^1,2^, Yun Han^1,2^, Fang Lai^1,2,3^  1 The Second Affiliated Hospital of Guangzhou University of Chinese Medicine, Guangzhou, Guangdong, China.  2 Guangdong Provincial Hospital of Chinese Medicine, Guangzhou, Guangdong, China.  3 Doctoral student of the Second Clinical College of Guangzhou University of Chinese Medicine, Guangzhou, Guangdong, China.  4 Foshan Hospital of Traditional Chinese Medicine, Foshan, Guangdong, China. (P1)  Authors’ contributions : Fang Lai, Ruifeng Zeng and Yi Zheng drafted this manuscript; Fang Lai, Ruifeng Zeng and Yun Han designed the described study; Rongrong Fan, Yan Zhang, Yi Zheng and Gengbiao Zhou acquire data; Shutao Mai and Yan Zhang conduct the study; Fang Lai and Dongping Xie made statistical analysis; Yanna Weng and Jiongdong Du monitor the process of trial implementation and give constructive suggestions; all authors read and approved the final manuscript. (P16-17) |
|  | 5b | Name and contact information for the trial sponsor  Guangdong Science and Technology Department, 171 Lianxin Road, Yuexiu District, Guangzhou 510033, China  National Natural Science Foundation of China, 83 Shuangqing Road, Haidian District, Beijing 100085, China |
|  | 5c | Role of study sponsor and funders, if any, in study design; collection, management, analysis, and interpretation of data; writing of the report; and the decision to submit the report for publication, including whether they will have ultimate authority over any of these activities  Funders of this study have no role in any abovementioned activities. |
|  | 5d | Composition, roles, and responsibilities of the coordinating center, steering committee, end point adjudication committee, data management team, and other individuals or groups overseeing the trial, if applicable (see item 21a for DMC)  Fang Lai, Ruifeng Zeng and Yun Han designed the described study; Rongrong Fan, Yan Zhang, Yi Zheng and Gengbiao Zhou acquire data; Shutao Mai and Yan Zhang conduct the study; Fang Lai and Dongping Xie made statistical analysis; Yanna Weng and Jiongdong Du monitor the process of trial implementation and give constructive suggestions. The investigation is conducted in accordance with national laws and the Declaration of Helsinki principles and received oversees from Guangdong Provincial Hospital of Chinese Medicine Institutional Review Board and Scientific Research Department of Guangdong Provincial Hospital of Chinese Medicine. (P15 to P17) |
| **Introduction** | | |
| Background and rationale | 6a | Description of research question and justification for undertaking the trial, including summary of relevant studies (published and unpublished) examining benefits and harms for each intervention  Sepsis is a focus of the global health problem a balanced control of immune response against invading pathogens between the hyper and the hypo-inflammatory phase might be a potential promising treatment in sepsis. The present pilot, randomized, controlled study has been designed to evaluate the feasibility of *SNT* on immune function in sepsis patients. (P3 to P4) |
|  | 6b | Explanation for choice of comparators  We choose saline as the comparator in this study to equalize the effect of fluid supplement in severe sepsis. (P7) |
| Objectives | 7 | Specific objectives or hypotheses  The purpose of this pilot study is to test the feasibility of *SNT* on immune function of sepsis. (P4) |
| Trial design | 8 | Description of trial design, including type of trial (e.g., parallel group, crossover, factorial, single group), allocation ratio, and framework (e.g., superiority, equivalence, noninferiority, exploratory)  This is a single center prospective parallel randomized controlled study carried out at a tertiary hospital medical ICU in Guangzhou, embedded with a observational study of basic immune function evaluation of severe sepsis patients. Fifty eligible sepsis patients will be randomly divided 1:1 into the intervention or control group. (P4) |
| **Methods** | | |
| Participants, interventions, and outcomes | | |
| Study setting | 9 | Description of study settings (e.g., community clinic, academic hospital) and list of countries where data will be collected. Reference to where list of study sites can be obtained  This single center clinical study will be carried out in Guangdong Provincial Hospital of Chinese Medicine located at No.36 Yongan Street, Guangzhou 510145. (P4) |
| Eligibility criteria | 10 | Inclusion and exclusion criteria for participants. If applicable, eligibility criteria for study centers and individuals who will perform the interventions (e.g., surgeons, psychotherapists)  Inclusion criteria are (1) age: ≥18 and ≤85; (2) documented or suspected infection; (3) two of the following induced by infection: ①rectal temperature ＞38℃ or ＜36℃; ②HR ＞90bpm; ③RR ＞20bpm or pCO2＜32mmHg, or requiring ventilation; ④WBC count＞12×109/L or ＜4×109/L or immature forms of WBC ＞10%; (4) one of the following: ①arterial hypotesion (SBP＜90mmHg, MAP＜65mmHg, or an SBP decrease ＞40mmHg in adults or vasopressor-dependent to maintain SBP≥90mmHg or MAP≥65mmHg) persists for one hour or more in spite of adequate fluid resuscitation; ②OI≤300; ③pH≤7.30 or BE≤-5.0mmo/L with hyperlactatemia (≥3mmol/L); ④acute oliguria (urine output＜0.5mg/kg/h for at least two hours), creatinine increase ＞2mg/dl within 48 hours or requiring RRT; ⑤hyperbilirubinemia (plasma total bilirubin ＞2mg/dL or 35umol/L); ⑥platelet count ＜10×1012/L; ⑦INR＞1.5 or APTT＞60s; (5) Yin syndrome in syndrome differentiation (according to principles of traditional Chinese medicine syndrome differentiation by two associate chief or above physicians respectively).  Exclusion criteria are (1) pregnant or lactating women; (2) patients receiving immunosuppressive or immunoenhancement therapy in the past 3 months; (3) patients with known or suspicious autoimmune diseases; (4) patients not expected to survive 28 days due to end-stage disease or other uncorrectable medical condition; (5) fasting subjects; (6) known or suspicious allergy to any ingredient of *SNT*. (P5 and Table 1) |
| Interventions | 11a | Interventions for each group with sufficient detail to allow replication, including how and when they will be administered  Consenting eligible patients will be randomized to receive *SNT* (*SNT* group, 150ml, po, qd, for 3 days) or equivalent volume of normal saline (control group), other than which articipants in both arms will receive standard therapy according to the SSC International Guidelines recommendations.  *SNT* (composed by 15g of *Aconitum carmichaelii,* 9g of *Debeaux, Zingiber officinale Roscoe*, and 6g of *Glycyrrhiza uralensis Fisch*) used in the study is manufactured and offered by the pharmaceutical department of Chinese herbal medicine of Guangdong Provincial Hospital of Chinese Medicine. (P7) |
|  | 11b | Criteria for discontinuing or modifying allocated interventions for a given trial participant (e.g., drug dose change in response to harms, participant request, or improving/worsening disease)  Participants are allowed to withdraw from the trial for any reason at any time. Researchers are able to remove participants from the trial to ensure their safety or maintain the quality of the trial. Participants with any of the following conditions may be removed from the study: 1. severe complications and/or general health deterioration occur; 2. Violating the study protocol; 3. withdrawal from the trial or loss to follow-up voluntarily. (P5) |
|  | 11c | Strategies to improve adherence to intervention protocols, and any procedures for monitoring adherence (e.g., drug tablet return, laboratory tests)  Investigators carrying out the experiment will be trained beforehand and provided a printed standardized protocol and CRF. The process of trial implementation is monitored by Yanna Weng and Jiongdong Du. (P7) |
|  | 11d | Relevant concomitant care and interventions that are permitted or prohibited during the trial  Concomitant medications as the SSC recommended are permitted, while immunosuppressive or immunoenhancement therapy is prohibited. (P7) |
| Outcomes | 12 | Primary, secondary, and other outcomes, including the specific measurement variable (e.g., systolic blood pressure), analysis metric (e.g., change from baseline, final value, time to event), method of aggregation (e.g., median, proportion), and time point for each outcome. Explanation of the clinical relevance of chosen efficacy and harm outcomes is strongly recommended  The primary endpoint is the feasibility of the study, in order to: (1) explore the feasibility for recruiting, randomizing and retaining participants, (2) evaluate acceptability and compliance of Si-Ni-Tang as an additional intervention of usual care, (3) evaluate outcome measures’ appropriateness, (4) generate data for effect size calculation in future sample size calculating, and (5) develop an appropriate protocol for further study. The length of time for recruitment of 50 eligible patients, recruitment rate and dropout rate will be measured. Successful recruitment is defined as at least half (50%) of eligible patients enrolled, with a dropout rate no more than 20%. The secondary endpoints include 28-day all cause mortality rates, immune function parameters, inflammation parameters, SOFA scores and APACHE II scores. The exploratory outcomes are ICU mortality, ICU length of stay, hospital mortality, and hospital length of stay. Safety outcomes includes CBC, general urine analysis, CRP, liver function tests (ALT, AST, ALP, TBIL, GGT), renal function test (BUN, Scr), which will be performed at Day 0 and Day 3. (P9) |
| Participant timeline | 13 | Time schedule of enrollment, interventions (including any runins and washouts), assessments, and visits for participants. A schematic diagram is highly recommended (Figure).  SPIRIT timeline of measurements is listed in Figure 2. (P10) |
| Sample size | 14 | Estimated number of participants needed to achieve study objectives and how it was determined, including clinical and statistical assumptions supporting any sample size calculations  Not applicable for this pilot trial. |
| Recruitment | 15 | Strategies for achieving adequate participant enrollment to reach target sample size  Close collaboration between physicians and researchers in Intensive Care Unit Departments. At least one researcher of the study group will be noticed with every potential eligible participant to initiate the standard recruitment protocol. (P6) |
| Assignment of interventions (for controlled trials) | | |
| Allocation Sequence generation | 16a | Method of generating the allocation sequence (e.g., computer-generated random numbers), and list of any factors for stratification. To reduce predictability of a random sequence, details of any planned restriction (e.g., blocking) should be provided in a separate document that is unavailable to those who enroll participants or assign interventions.  Eligible patients enrolled into the interventional part are being randomly assigned to either *SNT* group or control group (1:1 ratio). The randomization is carried out by SPSS 17.0 with a block randomization size of four. The assignments of treatment are balanced with 2 participants for each group respectively in each block. Randomization assignment is carried out by the physician (Ruifeng Zeng) not involved the treatment and the follow-up assessment. (P6) |
| Allocation concealment mechanism | 16b | Mechanism of implementing the allocation sequence (e.g., central telephone; sequentially numbered, opaque, sealed envelopes), describing any steps to conceal the sequence until interventions are assigned  Allocation will be sequentially numbered stored in sealed envelopes until interventions assigned. (P6) |
| Implementation | 16c | Who will generate the allocation sequence, who will enroll participants, and who will assign participants to interventions  Ruifeng Zeng will generate the allocation sequence, Yan Zhang and Shutao Mai will enroll participants and assign participants to interventions according to pre-prepared allocation sequence. (P6) |
| Blinding (masking) | 17a | Who will be blinded after assignment to interventions (e.g., trial participants, care providers, outcome assessors, data analysts), and how  The laboratory technicians and the biostatisticians responsible for the statistical analysis will be blinded to the assigned treatments. (P6) |
|  | 17b | If blinded, circumstances under which unblinding is permissible, and procedure for revealing a participant’s allocated intervention during the trial  There is no blinding for treating physicians or participants in this trial, only the laboratory technicians and the biostatisticians responsible for the statistical analysis will be blinded to the assigned treatments. Therefore, no unblinding is needed under any circumstance. (P6) |
| Data collection, management, and analysis | | |
| Data collection methods | 18a | Plans for assessment and collection of outcome, baseline, and other trial data, including any related processes to promote data quality (e.g., duplicate measurements, training of assessors) and a description of study instruments (e.g., questionnaires, laboratory tests) along with their reliability and validity, if known. Reference to where data collection forms can be found, if not in the protocol.  Researchers are trained to collect trial data carefully according to standard protocol. Molecular biomarkers will be measured triplicated to assure quality. Data will ultimately be input into an electronic database (REDCap 5.7.0) designed for the study. Data entered into the database will be check by different researcher. All collected data forms will be kept in the hospital Scientific Research Department. (P11) |
|  | 18b | Plans to promote participant retention and complete follow-up, including list of any outcome data to be collected for participants who discontinue or deviate from intervention protocols  Follow-up phone call will be conducted to complete follow-up. (P10) |
| Data management | 19 | Plans for data entry, coding, security, and storage, including any related processes to promote data quality (e.g., double data entry; range checks for data values). Reference to where details of data management procedures can be found, if not in the protocol.  Data is entered by two researchers independently into the database, and will be checked by different trained researchers separately. (P11) |
| Statistical methods | 20a | Statistical methods for analyzing primary and secondary outcomes. Reference to where other details of the statistical analysis plan can be found, if not in the protocol.  The length of time for recruitment of 50 eligible patients, recruitment rate and dropout rate will be measured. Successful recruitment is defined as at least half (50%) of eligible patients enrolled, with a dropout rate no more than 20%. The recruitment rate is determined by the percentage of eligible patients who agree to participate during the recruitment phase of the study. The dropout rate is calculated by the percentage of patients who are lost after enrollment to the 28th day from enrollment with no outcome data collected. (P10)  Quantitative numerical results with normal distribution are presented as mean±SD, while non-normal distribution ones are presented as median and interquartile range. Categorical variables are expressed as frequencies or number. Equality of variances between groups is assessed by Levene’s test. Unpaired Student’s t-test is used for normal distributed quantitative variables, while the Wilcoxon nonparametric statistic is used for non-normally distributed ones. The Pearson’s chi-square test or Fisher exact test is used for categorical data when appropriate. Differences in biological parameters between arms over time will be evaluated by repeat measure of General Linear Model as appropriate. Enrolled subjects that are not able to continue the study or follow up after treatment will remain to perform an “intention to treat” analysis.  A probability value of P＜0.05 will be considered as statistical significant. All statistical analysis is performed with SPSS (version 17.0, Chicago, USA) for Windows by researchers not involved in implementing the trial. (P12) |
|  | 20b | Methods for any additional analyses (e.g., subgroup and adjusted analyses)  Not applicable for this pilot trial. |
|  | 20c | Definition of analysis population relating to protocol nonadherence (e.g., as-randomized analysis), and any statistical methods to handle missing data (e.g., multiple imputation)  Enrolled subjects that are not able to continue the study or follow up after treatment will remain to perform an “intention to treat” analysis. (P12) |
| Monitoring | | |
| Data monitoring | 21a | Composition of DMC; summary of its role and reporting structure; statement of whether it is independent from the sponsor and competing interests; and reference to where further details about its charter can be found, if not in the protocol. Alternatively, an explanation of why a DMC is not needed.  Data and safety will be monitored by Guangdong Provincial Hospital of Chinese Medicine Institutional Review Board and Scientific Research Department of Guangdong Provincial Hospital of Chinese Medicine, which are independent from the sponsor with no competing interests. (P12) |
|  | 21b | Description of any interim analyses and stopping guidelines, including who will have access to these interim results and make the final decision to terminate the trial  Not applicable for this pilot trial. |
| Harms | 22 | Plans for collecting, assessing, reporting, and managing solicited and spontaneously reported adverse events and other unintended effects of trial interventions or trial conduct  Any adverse event occurred to participants after enrollment to 28^th^ day from enrollment, no matter whether it is related to intervention of the study, will be recorded. Severe adverse event and unexpected adverse event will be reported to the Ethics Committee within 2 days. Intervention will be suspended and symptomatic treatment will be offered when needed. The principle investigator will decide whether the patients should discontinue participation. (P11 to P12) |
| Auditing | 23 | Frequency and procedures for auditing trial conduct, if any, and whether the process will be independent from investigators and the sponsor  The process of trial implementation is monitored at least once per 2 weeks by Yanna Weng and Jiongdong Du, who are independent from participants’ enrollment and data analyzed. (P7) |
| **Ethics and dissemination** | | |
| Research ethics approval | 24 | Plans for seeking REC/IRB approval  Ethics approved from the Guangdong Provincial Hospital of Chinses Medicine Institutional Review Board (approval number B2016-010.2-01). (P15) |
| Protocol amendments | 25 | Plans for communicating important protocol modifications (e.g., changes to eligibility criteria, outcomes, analyses) to relevant parties (e.g., investigators, RECs/IRBs, trial participants, trial registries, journals, regulators)  If there are important protocol changes, a new version of protocol will be submitted to Guangdong Provincial Hospital of Chinses Medicine Institutional Review Board. (P7) |
| Consent or assent | 26a | Who will obtain informed consent or assent from potential trial participants or authorized surrogates, and how (see item 32)  Yan Zhang and Shutao Mai will obtain informed consent from potential trial participants. (P6) |
|  | 26b | Additional consent provisions for collection and use of participant data and biological specimens in ancillary studies, if applicable  Not applicable for this pilot trial. |
| Confidentiality | 27 | How personal information about potential and enrolled participants will be collected, shared, and maintained in order to protect confidentiality before, during, and after the trial  Participants receive a participation ID in this study, with which personal information of participants is labeled in papers. Only investigators involved in this study have access to reference ID identity on an as-needed basis. (P10) |
| Declaration of interests | 28 | Financial and other competing interests for principal investigators for the overall trial and each study site  None. (P16) |
| Access to data | 29 | Statement of who will have access to the final trial data set, and disclosure of contractual agreements that limit such access for investigators  Fang Lai and Yun Han are responsible for the data and have the final dataset. (P16) |
| Ancillary and post-trial care | 30 | Provisions, if any, for ancillary and post-trial care, and for compensation to those who suffer harm from trial participation  Not applicable. |
| Dissemination policy | 31a | Plans for investigators and sponsor to communicate trial results to participants, health care professionals, the public, and other relevant groups (e.g., via publication, reporting in results databases, or other data-sharing arrangements), including any publication restrictions  Trial results will be reported to Guangdong Science and Technology Department to publish to the public. Also, the investigators plan to publish the results in journals. Participants can reach out for information of the study progress and any related information. (P16) |
|  | 31b | Authorship eligibility guidelines and any intended use of professional writers  Not applicable. |
|  | 31c | Plans, if any, for granting public access to the full protocol, participant-level data set, and statistical code  Not applicable. |
| **Appendices** | | |
| Informed consent materials | 32 | Model consent form and other related documentation given to participants and authorized surrogates  Model consent form and other related documentation given to participants and authorized surrogates are available from the corresponding author on reasonable request. (P1) |
| Biological specimens | 33 | Plans for collection, laboratory evaluation, and storage of biological specimens for genetic or molecular analysis in the current trial and for future use in ancillary studies, if applicable  Blood samples from participants will be collected and stored at -80℃ for the final analysis. (P11) |
